# Time-restricted feeding reveals a role for neural respiratory clocks in optimizing daily ventilatory-metabolic coupling in mice

**DOI:** 10.1152/ajpendo.00111.2024

**Published:** 2024-06-05

**Authors:** Aaron A. Jones, Gabriella M. Marino, Deanna M. Arble

**Affiliations:** Department of Biological Sciences, Marquette University, Milwaukee, Wisconsin, United States

**Keywords:** BMAL1, breathing, circadian rhythm, metabolic rate, Phox2b

## Abstract

The master circadian clock, located in the suprachiasmatic nuclei (SCN), organizes the daily rhythm in minute ventilation (V̇e). However, the extent that the daily rhythm in V̇e is secondary to SCN-imposed O_2_ and CO_2_ cycles (i.e., metabolic rate) or driven by other clock mechanisms remains unknown. Here, we experimentally shifted metabolic rate using time-restricted feeding (without affecting light-induced synchronization of the SCN) to determine the influence of metabolic rate in orchestrating the daily V̇e rhythm. Mice eating predominantly at night exhibited robust daily rhythms in O_2_ consumption (V̇o_2_), CO_2_ production (V̇co_2_), and V̇e with similar peak times (approximately ZT18) that were consistent with SCN organization. However, feeding mice exclusively during the day separated the relative timing of metabolic and ventilatory rhythms, resulting in an approximately 8.5-h advance in V̇co_2_ and a disruption of the V̇e rhythm, suggesting opposing circadian and metabolic influences on V̇e. To determine if the molecular clock of cells involved in the neural control of breathing contributes to the daily V̇e rhythm, we examined V̇e in mice lacking BMAL1 in Phox2b-expressing respiratory cells (i.e., BKOP mice). The ventilatory and metabolic rhythms of predominantly night-fed BKOP mice did not differ from wild-type mice. However, in contrast to wild-type mice, exclusive day feeding of BKOP mice led to an unfettered daily V̇e rhythm with a peak time aligning closely with the daily V̇co_2_ rhythm. Taken together, these results indicate that both daily V̇co_2_ changes and intrinsic circadian time-keeping within Phox2b respiratory cells are predominant orchestrators of the daily rhythm in ventilation.

**NEW & NOTEWORTHY** The master circadian clock organizes the daily rhythm in ventilation; however, the extent that this rhythm is driven by SCN regulation of metabolic rate versus other clock mechanisms remains unknown. We report that metabolic rate alone is insufficient to explain the daily oscillation in ventilation and that neural respiratory clocks within Phox2b-expressing cells additionally optimize breathing. Collectively, these findings advance our mechanistic understanding of the circadian rhythm in ventilatory control.

## Graphical abstract

**Figure:**
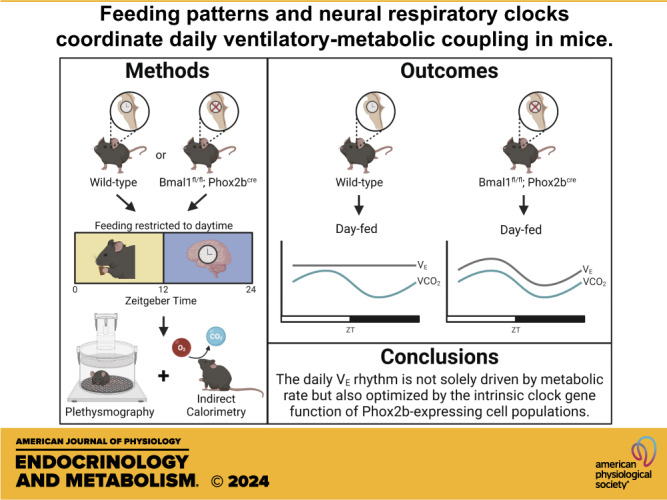

## INTRODUCTION

Most animals have developed a daily rhythm in minute ventilation (V̇e) to manage predictable daily variations in metabolic demand and vigilance state (1). Dysregulation of daily ventilatory rhythms has been implicated in a variety of respiratory diseases with time-specific symptoms including sleep apnea, sudden unexpected death in epilepsy, chronic asthma, obstructive pulmonary disease, and COVID-19 (1–5) . The daily rhythm in V̇e peaks during the active phase in rodents (6–9) and humans (10–11), but occurs independent of sleep-wake state (6) and activity level (12). This rhythm is endogenously driven by the master circadian clock in the brain, the suprachiasmatic nuclei (SCN), located in the anterior hypothalamus (7). However, the specific mechanisms by which the SCN organizes the daily rhythm in V̇e are not well understood.

Seminal studies suggest that metabolic rate (e.g., oxygen consumption and carbon dioxide production) is the primary determinant of the daily rhythm in V̇e because the ventilatory equivalent (i.e., V̇e normalized to metabolic rate) is typically constant across the day (6, 12). However, these early studies were conducted under standard light:dark cycles when metabolic rate is synchronized with endogenous circadian timing. Since oxygen consumption (V̇o_2_) and carbon dioxide production (V̇co_2_) are also organized by the endogenous circadian clock (13), it is unclear whether metabolic rate alone is sufficient to establish the daily rhythm in V̇e or if additional mechanisms controlled by the central circadian clock can independently regulate this rhythm. During reentrainment following brief light perturbation, ventilatory rhythms can phase-lead rhythms in V̇o_2_ and V̇co_2_ (14), suggesting ventilatory rhythms do not always occur secondary to metabolic rate. Moreover, by manipulating light duration to reorganize the circadian clock, we have previously demonstrated that the relationship between daily metabolic and ventilatory rhythms can be weakened (9). These studies indicate that the circadian clock may also influence ventilatory rhythms through mechanisms independent of daily V̇o_2_ and V̇co_2_ rhythms.

The SCN synchronizes time-keeping in all cells and tissues by organizing the expressional patterns of clock genes (15). One possibility is that the SCN optimizes ventilatory rhythms by synchronizing the molecular clocks of respiratory neurons directly. Recently, we demonstrated that disrupting the molecular clock of Phox2b-expressing cells via knockdown of the core clock gene BMAL1 attenuates circadian regulation of ventilatory chemoreflex (8). Phox2b-expressing cells are crucial for breathing due to their key role in sensing changes in O_2_ and CO_2_ (16–17) and other metabolic peptides/hormones (18–21). Others have demonstrated that Phox2b-expressing regions such as the nucleus tractus solitarius (NTS) of the dorsal respiratory group also exhibit strong circadian activity (21–23). Therefore, neural Phox2b-expressing cells are ideally positioned to integrate circadian and metabolic signals to optimize the daily rhythm V̇e during normal circadian alignment. However, the extent that cellular clocks within the respiratory network coordinate with daily metabolic rhythms to influence breathing remains unknown.

Here, we use a time-restricted feeding protocol to investigate the role of V̇o_2_ and V̇co_2_ cycles in shaping the daily rhythm in V̇e in mice. Feeding during the inactive phase causes a misalignment between food- and light-driven rhythms (13, 24) and is an established technique to separate metabolic rhythms from central circadian timing. Previous studies demonstrate that feeding-fasting rhythms shape daily cycles in V̇o_2_ and V̇co_2_ (13). Indeed, exclusive day-feeding of nocturnal rodents shifts metabolic rhythms toward the timing of food intake (13) without affecting signals derived from the SCN (24–25), allowing for experimental separation of metabolic rate from the light-dark cycle. Here we present evidence that exclusive day feeding disrupts the organization and daily phase-coupling of ventilatory and metabolic rhythms, indicating that mechanisms beyond metabolic rate alone contribute to the daily organization of V̇e in wild-type mice. We further demonstrate that disruption of the molecular clock specifically within Phox2b-expressing cells mitigates the dissociation of these rhythms under exclusive day-feeding. We conclude that the daily rhythm in V̇e is organized by metabolic rate as well as other circadian mechanisms such as clock gene expression within the neural respiratory network.

## METHODS

All methods were reviewed, approved by, and performed according to the guidelines of the Institutional Animal Care and Use Committee of Marquette University (Milwaukee, WI).

### Animals

All mice used in the present study were on a C57Bl/6J background and purchased from the Jackson Laboratory. Male and female mice 22 wk old (Strain No. 000664) were used for wild-type studies. F2 generation (15–37 wk old) male and female BKOP mice (BMAL1^fl/fl^;Phox2b^cre/+^) and littermate controls (i.e., BMAL1^fl/fl^;Phox2b^+/+^ and BMAL1^+/+^;Phox2b^cre/+^) were bred and genotyped in house as previously described (8). All mice were single-housed. A 21% kcal/fat chow diet (PicoLab Mouse Diet 20) and water were provided ad libitum until the start of the time-restricted feeding protocol. For all experiments, mice between groups were age- and weight-matched for all measurements.

### Time-Restricted Feeding

At the start of the experiment, a subset of mice remained on the chow diet (*n* = 8), while the remaining mice (*n* = 24) were switched to a 45% kcal/fat high-fat diet (HFD; Teklad TD.06415). After adjusting HFD-fed mice (*n* = 24) to the new diet for 3 days, all mice were fasted for 24 h to aid with reentrainment of food rhythms and then randomly assigned to one of three feeding schedules: ad libitum (*n* = 8), day-fed (*n* = 8), or night-fed (*n* = 8). The more palatable high-fat diet was implemented in these three subsequent groups to promote sufficient food intake in the day-fed group, allowing for experimental shifting of metabolic rhythms. Mice that were day-fed were allowed HFD food access during the entire light phase occurring between ZT 0 and 12. Mice that were night-fed were allowed HFD food access during the entire dark phase occurring between ZT 12 and 24. Food was time-restricted manually by switching mice between two habituated home cages: one with and one without food as previously described (26). Ad libitum mice were switched between two food-containing cages as a control. The body weight and food intake of wild-type mice were measured weekly during time-restricted feeding (TRF) for 4 wk. All mice stayed on a 12-h light:12-h dark cycle for the duration of the experiment. BKOP mice followed a similar protocol but were distributed only into day-fed and night-fed HFD groups.

### Ventilatory Measurements

During weeks 2 or 3 of TRF, mice had their 24-h breathing measured using whole body plethysmography (SCIREQ/Emka Technologies, Paris, France). After a 1-h acclimation period, mice remained inside the chamber for 24 h and were measured in real-time for ventilatory measures including tidal volume, respiratory rate, and V̇e while being exposed to room air (21% O_2_, 79% N_2_, 0% CO_2_) at a flow rate of 0.5 L/min. Pressure signals were adjusted for temperature and humidity changes using a volume correction factor, calculated using the Drorbaugh and Fenn equation (27), and applied on a breath-by-breath basis. IOX 2.1 software was used to generate real-time ventilatory measures. Threshold requirement settings were used as previously described (9). Data were averaged into 30-min bins to create a 24-h breathing profile. Food and water were provided in the chambers during data collection, and food was time-restricted manually for day- and night-fed groups by adding or removing food pellets from the chambers during the light transitions.

### Metabolic Assessment

During *week 3* or *4* of TRF, mice were transferred to Promethion indirect calorimetry cages (Sable Systems, North Las Vegas, NV) to assess daily patterns in metabolic gas exchange. All mice were acclimated to the cages for three continuous LD cycles while being maintained on their TRF schedule, and metabolic data were collected on the fourth cycle. While in the cages, food was time-restricted using automated food access doors. V̇o_2_ and V̇co_2_ rates were continuously recorded and averaged into 15-min bins to create 24-h profiles. Food intake data were averaged into 30-min bins to construct a 24-h profile of food activity.

### Immunohistochemistry

BMAL1 and Phox2b expression was assessed in the dorsal medullary brainstem via immunohistochemistry. Animals were euthanized at ZT 2 via inhaled CO_2_ exposure and decapitation. Brains were quickly harvested and fixed in chilled 4% paraformaldehyde for 24 h and then immersed in 30% sucrose for at least 48 h. After fixation, brains were placed into optimal cutting temperature (OCT) solution and stored at −20°C until use. A series of 25 μm sections from the brainstem were obtained with a Leica CM1900 cryostat. Tissue sections were washed three times in 0.1% Triton in PBS and then immersed in a blocking buffer (0.3% PBST with 0.3 M glycine and 5% donkey serum) for 20 min at room temperature. Brain sections were incubated at room temperature overnight with the following primary antibodies: rabbit anti-BMAL1 (1:500; No. 14020S, Cell Signaling) and goat anti-Phox2b (1:200; No. AF4940-SP, Novus Biologicals). After rinsing with 0.1% Triton in PBS, tissue sections were then incubated at room temperature for 2 h with the following secondary antibodies: donkey anti-rabbit conjugated with Alex Fluor 647 (1:1,000; No. A-31573, ThermoFisher) and donkey anti-goat conjugated with Alex Fluor 488 (1:1,000; No. A-11055, ThermoFisher). Finally, the sections were mounted on tissue-adhesive slides and imaged with a fluorescence microscope (Keyence, Itasca, IL). For colocalization analysis, brainstem slices from the approximate coordinates −2.68 (interaural), −6.48 (bregma) were used. Colocalization of BMAL1 and Phox2b was analyzed using QuPath v0.5.1 software.

### Statistical Methods

All data are presented as the means ± SE. Group differences were tested as appropriate using *t* test, one-way, two-way, or three-way analysis of variance (ANOVA) with post hoc tests and repeated measures as indicated using GraphPad Prism 9. Rhythmicity and relative peak timing (i.e., center of gravity) of food intake, V̇o_2_, V̇co_2_, and V̇e were determined by cosinor fit analysis using CircWave V1.4 software (28).

## RESULTS

### Exclusive Day Feeding Shifts Daily Rhythms in Metabolic Rate toward the Timing of Food Intake

**Figure 1.**
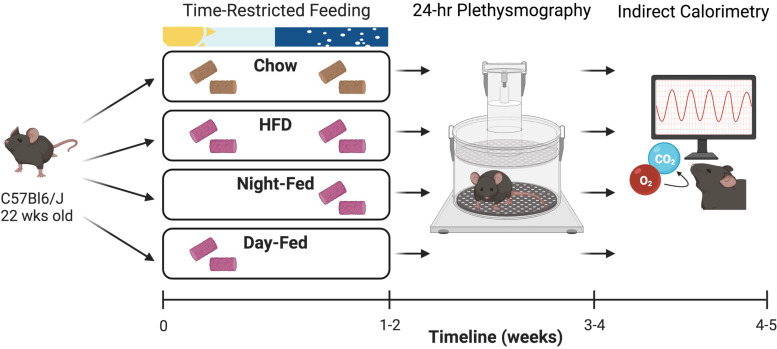
Time-restricted feeding protocol. Wild-type C57Bl6/J mice were randomly distributed into four TRF groups: chow-fed ad libitum (Chow), HFD-fed ad libitum (HFD), HFD night-fed, or HFD day-fed, and maintained on their respective feeding schedules for 4 wk. Ventilation was assessed during *weeks 2* and *3* of TRF using whole body plethysmography. Metabolic rate was assessed during *weeks 3* and *4* of TRF using indirect calorimetry. TRF, time-restricted feeding. Created using Biorender.com.

Wild-type C57Bl6/J mice were randomly distributed into four TRF groups: chow-fed ad libitum (Chow), HFD-fed ad libitum (HFD), HFD day-fed (DF), or HFD night-fed (NF), and maintained on their respective feeding schedules for 4 wk (Fig. 1). Ventilatory and metabolic rhythms were assessed after mice were entrained to their respective feeding schedules for at least 1 wk. After 4 wk of TRF, there was no effect of feeding condition on body weight (Supplemental Fig. S1*A*; *P* = 0.79). Day-fed mice exhibited a decreased cumulative food intake compared with chow-fed mice (Supplemental Fig. S1*B*; *P* = 0.038) but not the other groups fed with a high-fat diet. All groups demonstrated a significant daily rhythm in food intake according to cosinor analysis (Supplemental Fig. S1*C*; *P* < 0.0001 for all groups).

**Figure 2.**
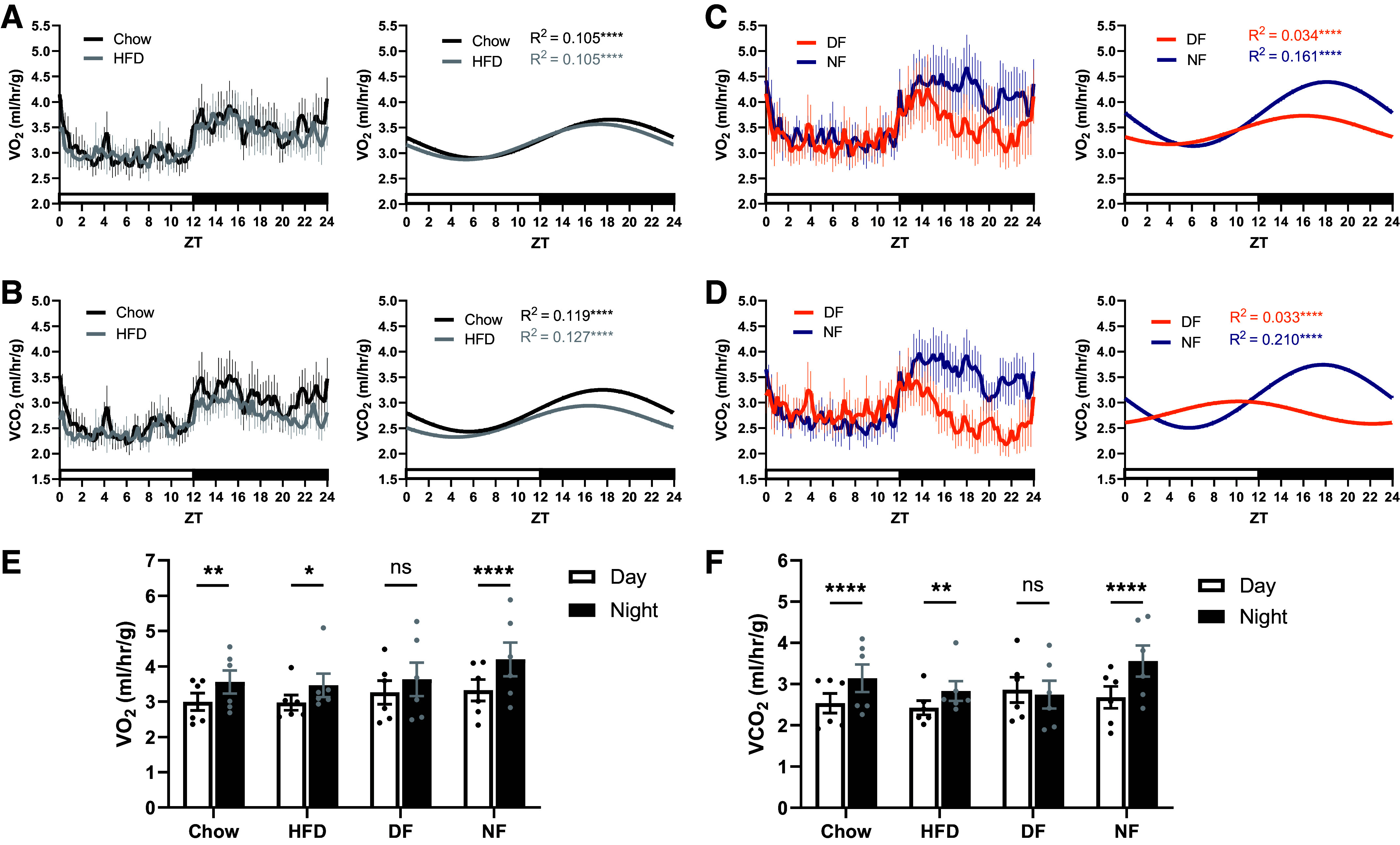
Day feeding shifts rhythms in metabolic rate toward the timing of food intake. Under chow-fed and HFD-fed conditions, V̇o_2_ (*A*) and V̇co_2_ (*B*) exhibited a significant daily rhythm. Under day-fed and night-fed conditions, V̇o_2_ (*C*) and V̇co_2_ (*D*) exhibited a significant daily rhythm. Mice exhibited significant day-night differences in V̇o_2_ (*E*) and V̇co_2_ (*F*) under all feeding conditions except day-fed. *n* = 6 for all groups (three male, three female). *A–D*: Cosinor fit analysis where α = 0.05. *E* and *F*: repeated measures two-way ANOVA with Sidak’s post hoc. *P < 0.05, **P < 0.01, ****P < 0.0001. HFD, high-fat diet; DF, day-fed; NF, night-fed; ns, not significant; V̇co_2_, carbon dioxide production; V̇o_2_, oxygen consumption; ZT, zeitgeber time.

Although female mice exhibited higher V̇o_2_ and V̇co_2_ overall per unit body weight (Supplemental Fig. S2; *P* < 0.001 for both) consistent with previous work (13), there were no interaction effects of TRF with sex (*P* = 0.92 for V̇o_2_; *P* = 0.91 for V̇co_2_) or TRF with sex and time of day (*P* = 0.99 for V̇o_2_; *P* = 0.55 for V̇co_2_). Therefore, data were pooled together with an evenly balanced number of males and females in each feeding group. Wild-type mice demonstrated robust daily rhythms in V̇o_2_ and V̇co_2_ under all feeding conditions (Fig. 2, *A*–*D*; *P* < 0.0001 for all groups). There were no significant differences in the relative peak times of V̇o_2_ (Fig. 2*A*; *P* = 0.97) or V̇co_2_ (Fig. 2*B*; *P* = 0.94) rhythms for mice fed chow or HFD ad libitum, suggesting that diet composition does not influence the daily timing of metabolic rate under our specific feeding protocol. Similarly, there were no differences in the relative peak times of metabolic rhythms in ad libitum HFD-fed versus exclusive night-fed groups (*P* = 0.99 for V̇o_2_; *P* = 0.93 for V̇co_2_), suggesting no considerable effect of the fasting experienced during time-restricted feeding on relative peak timing either. The relative peak time of V̇o_2_ (Fig. 2*C*) was not significantly affected by daytime feeding (*P* > 0.05 vs. Chow, HFD, and NF), despite a noticeable dampening of the rhythm. The relative peak time of V̇co_2_ (Fig. 2*D*) was significantly phase-advanced by approximately 8.5 h (*P* < 0.01 vs. Chow, HFD, and NF), toward the timing of food intake. We next examined the mean values of each variable during the 12-h day and night phases, separately. Mice in the day-fed group failed to exhibit day-night variation in V̇o_2_ (Fig. 2*E*; *P* = 0.10) and V̇co_2_ (Fig. 2*F*; *P* = 0.75). This is in contrast to chow-fed, HFD-fed, and night-fed mice, which all exhibited significant day-night variation in these variables (Fig. 2, *E* and *F*; *P* < 0.05 for all). The daily, 24-h means of V̇o_2_ (Supplemental Fig. S3*A*; *P* = 0.68) and V̇co_2_ (Supplemental Fig. S3*B*; *P* = 0.69) were not altered by time-restricted feeding. These results indicate that, in wild-type mice, the exclusive day feeding paradigm shifts metabolic rate toward the timing of food intake.

### Misalignment of V̇co_2_ with Central Circadian Timing under Exclusive Day Feeding Disrupts the Daily Rhythm in V̇e

**Figure 3.**
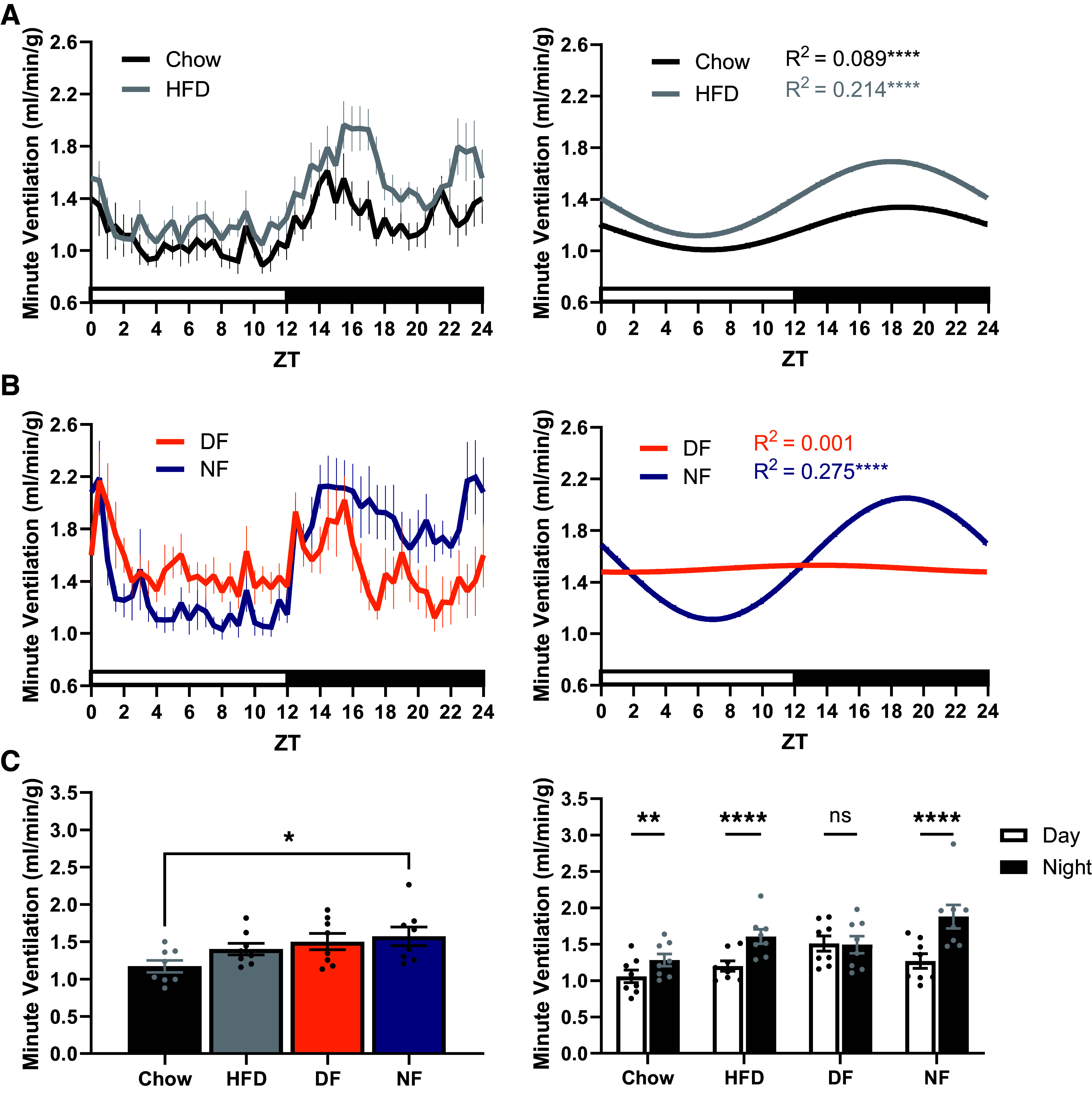
The daily rhythm in minute ventilation is disrupted under exclusive day feeding. *A*: minute ventilation exhibited a significant daily rhythm under chow-fed and HFD-fed conditions. *B*: minute ventilation exhibited a significant daily rhythm under night-fed but not day-fed conditions. *C*: the daily mean minute ventilation of night-fed mice was increased relative to chow-fed mice. Day-fed mice failed to exhibit significant day-night variation in minute ventilation. *n* = 8 for all groups (four males, four females). *A* and *B*: Cosinor fit analysis where α = 0.05. *C*: one-way ANOVA with Tukey’s post hoc (*left*) and repeated measures two-way ANOVA with Sidak’s post hoc (*right*). *P < 0.05, **P < 0.01, ****P < 0.0001. HFD, high-fat diet; DF, day-fed; NF, night-fed; ZT, zeitgeber time.

Consistent with our past observations (8), female mice exhibited a higher overall V̇e per unit body weight compared with males (Supplemental Fig. S2; *P* < 0.0001); however, there were no interaction effects of TRF with sex (*P* = 0.90) or TRF with sex and time of day (*P* = 0.89). Chow-fed, HFD-fed, and night-fed groups demonstrated robust daily rhythms in V̇e (Fig. 3, *A* and *B*; *P* < 0.0001 for each group) with similar peak times. However, exclusive day-fed mice had no detectable monophasic rhythm in V̇e (Fig. 3*B*; *P* = 0.76). Significance was detected when V̇e was fitted as a biphasic pattern (Supplemental Fig. S4; *P* < 0.0001), with one peak corresponding to the presentation of food and another corresponding to dark onset (i.e., environmental cues). Night-fed mice exhibited an increase in daily mean V̇e relative to chow-fed mice (*P* = 0.040), and all groups except day-fed mice (*P* = 0.99) exhibited significant day-night variation in V̇e (Fig. 3*C*; *P* < 0.01 for Chow, HFD, and NF). Collectively, these findings indicate that predominant feeding during the dark phase is required to maintain an organized monophasic rhythm in V̇e, and that V̇o_2_ or V̇co_2_ alone is insufficient to organize the daily V̇e rhythm under an exclusive day-feeding protocol.

### V̇co_2_ is Sufficient to Organize the Daily V̇e Rhythm of BKOP Mice

Brainstem pontomedullary regions modulate the respiratory pattern in response to changing O_2_ and CO_2_ levels to maintain blood gas homeostasis (29). Many of the neural cells involved in chemosensory ventilatory responses are derived from neural crest cells expressing the transcriptional marker Phox2b (30–31). Since mice lacking BMAL1 expression in Phox2b-expressing cells (BKOP) demonstrate time-specific changes in ventilatory chemoreflex (8), we reasoned that the molecular clock within Phox2b cells may play an integrative role in shaping the daily rhythm in V̇e by optimizing ventilatory adjustments to daily changes in O_2_ and CO_2_ levels. Therefore, we next determined whether rhythms in V̇e would shift with metabolic rate upon disrupting circadian gene expression within Phox2b-expressing cells.

In agreement with our previous work (8), we verified that BKOP mice exhibit a normal daily rhythm in V̇e under ad libitum feeding relative to control littermates (Supplemental Fig. S5; *P* = 0.89), despite having a markedly reduced number of BMAL1+/Phox2b+ cells in the dorsal respiratory group of the brainstem (Supplemental Fig. S6; *P* = 0.027). We next exposed BKOP mice to exclusive day feeding or exclusive night feeding to examine ventilatory and metabolic rhythms. Only the night-fed condition was used as a control in BKOP mice because the peak timing of metabolic and ventilatory rhythms in wild-type mice was similar between night-fed and ad libitum conditions. After 1 wk of TRF and just prior to ventilatory assessment, day-fed BKOP mice weighed similar to night-fed BKOP mice (Supplemental Fig. S7*A*; *P* = 0.56). During metabolic assessment, there was no statistical difference in total food intake between day-fed and night-fed BKOP mice (Supplemental Fig. S7*B*; *P* = 0.15). BKOP mice demonstrated a significant daily rhythm in food intake under both feeding conditions (Supplemental Fig. S7*C*; *P* < 0.0001 for both groups).

**Figure 4.**
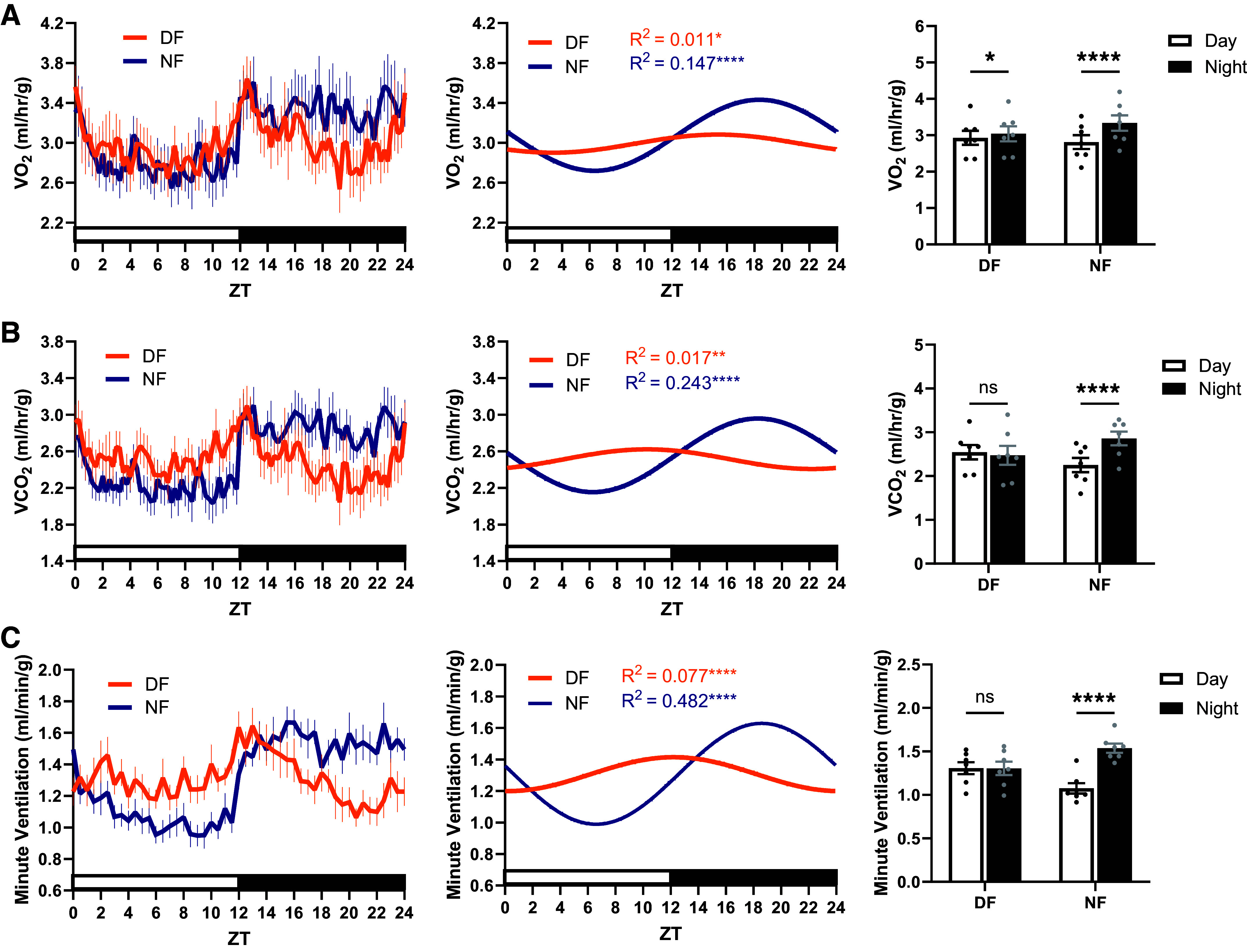
The daily rhythm in minute ventilation persists in BKOP mice fed exclusively during the day. *A*: BKOP mice exhibited a significant daily rhythm and day-night variation in V̇o_2_ under both feeding conditions. BKOP mice exhibited a significant daily rhythm in V̇co_2_ (*B*) and minute ventilation (*C*) under both feeding conditions and significant day-night variation under just the night-fed condition. *n* = 7 for all groups (five male, two female). *A*–*C*: Cosinor fit analysis where α = 0.05 (*left*) and repeated measures two-way ANOVA with Sidak’s post hoc (*right*). *P < 0.05, **P < 0.01, ****P < 0.0001. BKOP, BMAL1 knocked out of Phox2b cells, DF, day-fed; NF, night-fed; V̇co_2_, carbon dioxide production; V̇o_2_, oxygen consumption; ZT, zeitgeber time; ns, not significant.

Like wild-type mice, BKOP mice demonstrated significant daily rhythms in V̇o_2_ under both feeding conditions (Fig. 4*A*; *P* = 0.025 for DF, *P* < 0.0001 for NF). Both day-fed and night-fed BKOP mice exhibited significant day-night variation in V̇o_2_ (*P* = 0.029 for DF; *P* < 0.0001 for NF). Similarly, V̇co_2_ was rhythmic under day-fed and night-fed conditions (Fig. 4*B*; *P* = 0.0026 for DF, *P* < 0.0001 for NF). Night-fed (*P* < 0.0001), but not day-fed (*P* = 0.46), BKOP mice demonstrated significant day-night variation in V̇co_2_. Night-fed BKOP mice demonstrated a robust rhythm in V̇e (Fig. 4*C*; *P* < 0.0001). In addition, and in contrast to wild-type mice (Fig. 3*B*), BKOP mice exhibited a significant monophasic rhythm in V̇e under the day-fed condition (Fig. 4*C*; *P* < 0.0001). Night-fed (*P* < 0.0001), but not day-fed (*P* = 0.99), BKOP mice demonstrated significant day-night variation in V̇e. The daily means of V̇o_2_ (Supplemental Fig. S8*A*; *P* = 0.77), V̇co_2_ (Supplemental Fig. S8*B*; *P* = 0.86), and V̇e (Supplemental Fig. S8*C*; *P* = 0.99) were unaffected by feeding time in BKOP mice. These data suggest that clock function within Phox2b-expressing cells acts in opposition to metabolic rate when food intake and central circadian timing are misaligned, as knockdown of BMAL1 expression within this population reestablishes an unfettered daily rhythm in V̇e under day feeding.

### Day Feeding Elicits Phase Misalignment of Metabolic and Ventilatory Rhythms in Wild-Type Mice, but Not BKOP Mice

**Figure 5.**
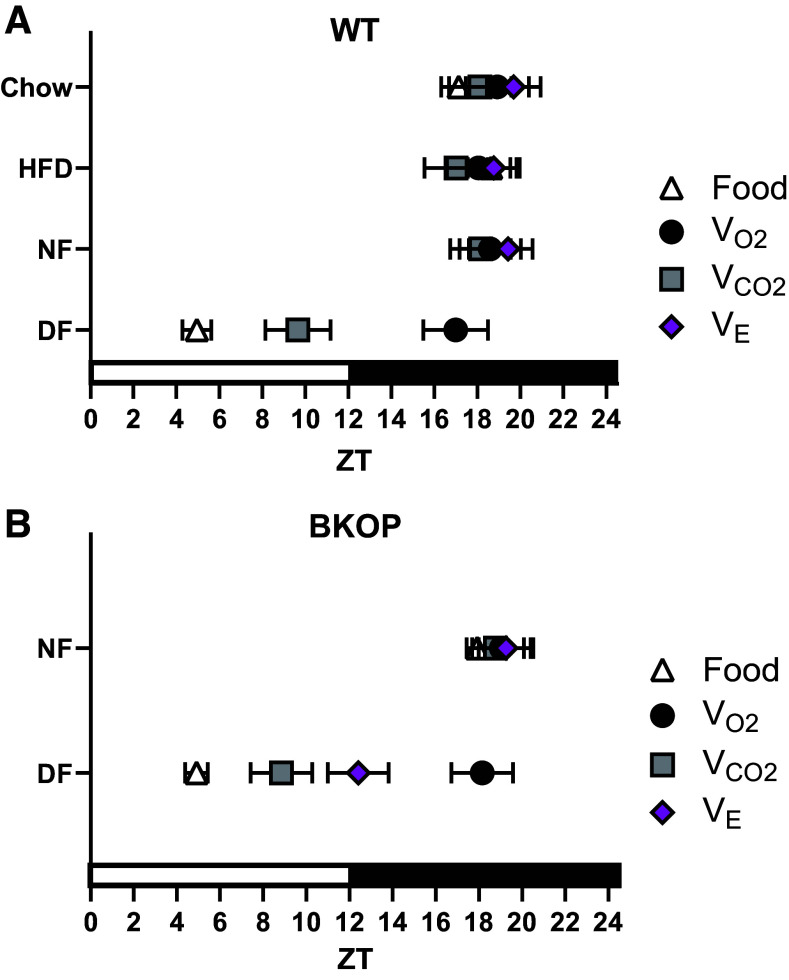
Day feeding differentially organizes the daily rhythm in minute ventilation of wild-type and BKOP mice. *A*: under all feeding conditions except day-fed, the phases of food intake, V̇o_2_, and V̇co_2_ (*n* = 6; three males, three females) in wild-type mice remained tightly aligned with the V̇e rhythm (*n* = 8; four males, four females) during the mid-dark phase. Under day feeding, V̇co_2_ shifted toward food intake (*P* < 0.01). *B*: in night-fed BKOP mice, the phases of food intake, V̇o_2_, V̇co_2_, and V̇e rhythms remained tightly aligned to mid-dark phase. Under day feeding, the phase of V̇e was significantly different from that of V̇o_2_ (*P* = 0.0083) but not V̇co_2_ (*P* = 0.18). *n* = 7 for BKOP mice (five males, two females). *A* and *B*: two-way ANOVA with Tukey’s post hoc. BKOP, BMAL1 knocked out of Phox2b cells; DF, day-fed; NF, night-fed; V̇co_2_, carbon dioxide production; V̇e, minute ventilation; V̇o_2_, oxygen consumption; WT, wild-type; ZT, zeitgeber time.

To further determine the respective influences of metabolic rate and circadian timing on the organization of the daily rhythm in V̇e, we examined the phase relationships between food intake, V̇o_2_, V̇co_2_, and V̇e rhythms under each feeding condition in wild-type and BKOP mice (Fig. 5). For chow-fed, HFD-fed, and night-fed wild-type mice, the relative peak timing of V̇e remained tightly aligned with that of food intake, V̇o_2_, and V̇co_2_ in the middle of the dark phase (Fig. 5*A*). V̇o_2_ was not significantly shifted by exclusive day feeding (*P* > 0.05 vs. Chow, HFD, and NF), while V̇co_2_ was advanced by approximately 8.5 h (*P* < 0.01 vs. Chow, HFD, and NF), toward the timing of food intake. The peak time of V̇e in day-fed wild-type mice was not plotted due to lack of a significant monophasic rhythm (i.e., no phase marker). Within the day-fed group, the relative peak time of V̇co_2_ was significantly different compared with V̇o_2_ (*P* = 0.0011). These data suggest that desynchronizing food intake from central circadian timing leads to a dissociation of V̇o_2_ and V̇co_2_ rhythms and a weakening of daily ventilatory-metabolic coupling in wild-type mice.

Similar to wild-type mice, BKOP mice maintained synchrony between metabolic and ventilatory rhythms under exclusive night feeding (Fig. 5*B*). The relative peak time of V̇e remained tightly aligned with that of food intake, V̇o_2_, and V̇co_2_ in the middle of the dark phase. In BKOP mice fed exclusively during the day, the relative peak time of the V̇co_2_ rhythm was phase-advanced by approximately 10 h (*P* < 0.0001), toward the timing of food intake. The relative peak time of V̇o_2_ remained unshifted in the day-fed group (*P* = 0.97), and minute ventilation was phase-advanced by approximately 7 h (*P* = 0.0009). Within the day-fed group, the relative peak time of V̇co_2_ was significantly different compared with V̇o_2_ (*P* < 0.0001). The relative peak time of V̇e under day feeding was significantly different compared with V̇o_2_ (*P* = 0.0083), but not V̇co_2_ (*P* = 0.18). These data indicate that preservation of the V̇e rhythm in day-fed BKOP mice is likely due to an unfettered association of breathing with the daily rhythm in V̇co_2_.

## DISCUSSION

Animals demonstrate a daily rhythm in V̇e that is controlled by the master circadian clock, the SCN (7). Over the course of the day, V̇e oscillates in parallel with V̇o_2_ and V̇co_2_ cycles (6, 12, 14), which are also SCN-controlled (13). However, the extent to which the V̇e rhythm is driven by SCN-imposed V̇o_2_ and V̇co_2_ cycles versus other SCN-derived mechanisms is unclear. Here, we used behavioral and transgenic approaches to demonstrate that the daily rhythm in V̇e is not solely driven by daily changes in metabolic rate but also optimized by intrinsic clock-derived mechanisms such as clock gene expression in the neural respiratory network.

Similar to previous findings (13), we found that day feeding was effective in phase-shifting the V̇co_2_ rhythm of wild-type animals by approximately 8.5 h toward the timing of food intake. However, we only measured an approximately 1.5-h adjustment to V̇o_2_ that was not statistically significant. The approximate 5-h shift in V̇o_2_ recorded by Adamovich and colleagues could be due to differences in animal age, diet composition (chow vs. HFD), and/or duration or time-restricted feeding prior to data collection. To our knowledge, the present study is the first to examine the daily rhythm in V̇e using altered feeding schedules. We found that the daily rhythm in V̇e of wild-type mice did not shift in accordance with V̇o_2_ or V̇co_2_ upon day feeding, but instead was eliminated. This finding is consistent with previous work demonstrating that the daily timing of V̇e does not adjust perfectly with V̇o_2_ in response to altered light:dark cycles (9, 14). Thus, neither V̇o_2_ nor V̇co_2_ rhythms appear sufficient to organize the daily V̇e rhythm of wild-type mice. Instead, central circadian timing likely plays an additional modulatory role. Loss of the V̇e rhythm under day feeding likely reflects opposing influences of metabolic (e.g., food) and central circadian (e.g., light) cues, implying that other clock-controlled signals may simultaneously adjust V̇e independent of metabolism. Interestingly, V̇e exhibited a biphasic pattern under day feeding, likely a consequence of environmental cues acutely driving behavior. Indeed, one peak corresponded with the presentation of food, while the other occurred just after dark onset. This observation is consistent with reports that the light environment can acutely regulate breathing independent of circadian timing (32–33).

Although we closely tracked daily organization of rhythms in food intake and metabolic gas exchange, this study is limited in that we cannot account for the partial contributions of other physiological rhythms to the disruption of the V̇e rhythm in day-fed wild-type mice. Day feeding increased mean V̇e during the light phase, which could be attributable in part to increased wakefulness during feeding rather than metabolic rate per se. Although the daily rhythm in V̇e occurs independent of sleep-wake cycles (6), vigilance state does have additive effects on breathing which may have contributed to the disrupted rhythm in V̇e. Similarly, we did not measure locomotor activity, which may partially contribute to disruption of the V̇e rhythm of wild-type mice in response to day feeding. Although the daily V̇e rhythm occurs independent of locomotor activity (7, 9, 12), day feeding only marginally affects locomotor activity (13). Indeed, timing of locomotor activity could mediate part of the “central” clock effects on the daily rhythm in V̇e. TRF can act as a zeitgeber for core body temperature (CBT) rhythms (34), disrupt normal body temperature homeostasis (35), and uncouple rhythms in CBT and metabolic rate (36). Thus, manipulating the timing of food intake without altering the light:dark cycle may exacerbate the magnitude of ventilatory rhythm disturbance by differentially affecting the timing of CBT and metabolic rate. However, it is worth highlighting that ventilatory rhythms correlate better with metabolic rate in humans, even when CBT is approximately 8 h phase-delayed relative to metabolic rate (10), suggesting that the endogenous CBT rhythm is less governing to the daily rhythm in V̇e, at least in humans.

Clock genes and genes involved in respiratory plasticity are rhythmically expressed in the dorsal and ventral areas of the brainstem (22, 37). The SCN may optimize the daily rhythm in V̇e through the organization of molecular clock rhythms within brainstem respiratory neurons. We therefore utilized a conditional BMAL1 knockout mouse in which the molecular clock is disrupted exclusively in Phox2b-expressing cells (i.e., BKOP), which are involved in chemoreception, sympathetic outflow, and tonic ventilatory drive (16, 30, 38). We found that BKOP mice exhibited similar rhythms in V̇co_2_ and V̇o_2_ compared with wild-type mice under day feeding. However, whereas wild-type mice lacked a monophasic rhythm in V̇e during exclusive day feeding, BKOP mice maintained a robust daily rhythm in V̇e under day feeding that was phase-advanced by approximately 7 h relative to night-fed controls. The rescued daily V̇e rhythm in day-fed BKOP mice aligned adequately with the relative peak timing of V̇co_2_, an outcome consistent with the fundamental principles of alveolar ventilation (39). This finding suggests that the elimination of the daily V̇e rhythm under day feeding in wild type mice is due to V̇co_2_ acting in opposition to the molecular clock of Phox2b-expressing cells, which is likely dictated by light-induced organization of the SCN. Consequently, disruption of the molecular clock within this population removes this opposing signal, allowing V̇co_2_ to sufficiently organize a now unfettered daily V̇e rhythm in BKOP mice.

Our previous work demonstrates that the molecular clock of Phox2b-expressing cells is an important regulator of ventilatory chemoreflexes (8). Although Phox2b-expressing cells act as canonical respiratory chemosensors for arterial O_2_ and CO_2_, and brain acidity (16–18, 29, 30, 40), the specific sensory mechanism for V̇co_2_ regulation of breathing remains elusive (38). However, others have suggested an integrative role for Phox2b-expressing regions (e.g., the RTN) in modifying ventilation in proportion to V̇co_2_ (38, 40, 41). Although we do not assert a role for Phox2b-expressing cells in directly sensing the V̇co_2_ stimulus per se, our results suggest that the SCN may synchronize the molecular clock of Phox2b-expressing cells to optimize other regulatory mechanisms that drive breathing in anticipation of situations requiring efficient linkage of V̇e and V̇co_2_, such as during food availability/intake or high metabolic demand (e.g., physical activity).

The precise Phox2b-expressing populations involved in daily ventilatory-metabolic coupling remain to be elucidated; however, the NTS may be the most promising candidate. The NTS has been implicated as a strong extra-SCN rhythmic controller of autonomic and metabolic regulation (23). Indeed, the NTS is a Phox2b-expressing region located in the dorsal respiratory group of the brainstem that functions in a circadian manner and integrates metabolic signals to modulate breathing (20–21). In BKOP mice, we observed a decrease in BMAL1-expressing cells within the presumptive NTS. Thus, the molecular clock of NTS neurons in particular may optimize the daily V̇e rhythm to predictable changes in V̇co_2_ across the 24-h day, warranting further exploration.

Despite BKOP mice demonstrating a robust daily rhythm in V̇e under day feeding, we cannot rule out the contributions of other clock-regulated mechanisms in optimizing the daily rhythm in V̇e. Of note, clock gene function exclusively in the respiratory rhythm generator itself has not been explored. Moreover, although clock genes are present in other respiratory structures such as the cervical spinal cord and diaphragm (37) and the lungs/airway (42–43), it remains unknown how clock gene function in these regions impacts daily regulation of V̇e.

Although female mice tend to exhibit overall higher V̇o_2_, V̇co_2_, and V̇e per unit body weight compared with males (8, 13), we found that the response of daily metabolic and ventilatory rhythms to feeding time did not differ based on biological sex in wild-type mice. This study is limited in that the small number of available BKOP females hinders our ability to examine sex differences in TRF response for BKOP mice. However, we have previously observed that the circadian V̇e rhythm of BKOP mice is indistinguishable from littermate controls in both male and females (8). Although we speculate that sex might not play a prominent role in the ventilatory response to feeding patterns, examination of sexual dimorphism in future studies investigating the circadian control of breathing is of great interest.

Metabolic disturbance is a common feature of circadian disruption (44). Circadian disruption exacerbates obesity and metabolic syndrome (45), making individuals more susceptible to hypoventilation, daytime hypercapnia, and sleep-disordered breathing (46–47). Our results suggest that feeding at the “wrong” time of day may lead to a partial separation in the daily rhythms in metabolic rate and V̇e. While it has yet to be tested, prolonged misalignment of metabolic and ventilatory rhythms could contribute to the respiratory insufficiency observed in humans experiencing persistent circadian disruption (48–49). In contrast, pairing food intake exclusively to the active phase (i.e., night feeding in nocturnal rodents) increases overall V̇e and leads to a more robust daily V̇e rhythm. Proper alignment of metabolic rate via timed meals to an individual’s active phase could conceivably provide therapeutic benefits for respiratory health. However, no studies to date have investigated the effects of TRF in the context of respiratory disease.

Collectively, these data demonstrate that the daily rhythm in V̇e is not merely secondary to SCN-driven patterns in metabolic rate. Instead, we find that central circadian timing synergizes with metabolism to optimize the daily rhythm in V̇e and that misalignment of these variables alters the daily V̇e rhythm. In addition, we find that clock gene regulation within Phox2b-expressing cells acts as one of the circadian mechanisms that fine-tunes the daily rhythm in V̇e. Further studies are needed to uncover other clock-driven mechanisms that shape daily ventilatory behavior. Taken together, these findings may have implications for humans experiencing circadian disruption and respiratory disease.

## DATA AVAILABILITY

The original contributions presented in the study are included in the article. Further inquiries can be directed to the corresponding author. Data will be made available upon reasonable request.

## SUPPLEMENTAL DATA

10.6084/m9.figshare.25681851 Supplemental Figs. S1–S8: https://doi.org/10.6084/m9.figshare.25681851.

## GRANTS

This study was supported by a Marquette University Research and Faculty Grant (to D.M.A.). A.A.J. was supported by the Marquette University Richard W. Jobling Distinguished Research Assistantship.

## DISCLOSURES

No conflicts of interest, financial or otherwise, are declared by the authors.

## AUTHOR CONTRIBUTIONS

A.A.J. and D.M.A. conceived and designed research; A.A.J. performed experiments; A.A.J. and G.M.M. analyzed data; A.A.J. and D.M.A. interpreted results of experiments; A.A.J. and G.M.M. prepared figures; A.A.J. drafted manuscript; A.A.J. and D.M.A. edited and revised manuscript; A.A.J., G.M.M., and D.M.A. approved final version of manuscript.
